# Genome-Edited T Cell Therapies

**DOI:** 10.1007/s40778-017-0077-5

**Published:** 2017-04-18

**Authors:** Juliette M. K. M. Delhove, Waseem Qasim

**Affiliations:** Molecular Immunology Unit, UCL Great Ormond Street Institute of Child Health, University College London (UCL), 30 Guilford Street, London, WC1N 1EH UK

**Keywords:** Genome editing, CRISPR/Cas9, T cell therapies, Chimeric antigen receptors, T cell receptors, Clinical trials, Immunotherapy

## Abstract

**Purpose of Review:**

Alternative approaches to conventional drug-based cancer treatments have seen T cell therapies deployed more widely over the last decade. This is largely due to their ability to target and kill specific cell types based on receptor recognition. Introduction of recombinant T cell receptors (TCRs) using viral vectors and HLA-independent T cell therapies using chimeric antigen receptors (CARs) are discussed. This article reviews the tools used for genome editing, with particular emphasis on the applications of site-specific DNA nuclease mediated editing for T cell therapies.

**Recent Findings:**

Genetic engineering of T cells using TCRs and CARs with redirected antigen-targeting specificity has resulted in clinical success of several immunotherapies. In conjunction, the application of genome editing technologies has resulted in the generation of HLA-independent universal T cells for allogeneic transplantation, improved T cell sustainability through knockout of the checkpoint inhibitor, programmed cell death protein-1 (PD-1), and has shown efficacy as an antiviral therapy through direct targeting of viral genomic sequences and entry receptors.

**Summary:**

The combined use of engineered antigen-targeting moieties and innovative genome editing technologies have recently shown success in a small number of clinical trials targeting HIV and hematological malignancies and are now being incorporated into existing strategies for other immunotherapies.

## Introduction

T cell-based immunotherapies aim to target and lyse antigen-positive cells without detrimental effects to healthy cells. Early approaches focused on adoptive cell therapy (ACT), involving collection of potent antitumor T cells, ex vivo expansion, and reinfusion in an autologous fashion [1]. More recently, T cells have been genetically altered to express modified αβ T cell receptors (TCRs) that confer higher affinity for tumor antigen [2]. These TCRs recognize cell-surface and intracellular processed peptides presented in the context of self-major histocompatibility complex (MHC). Alternative synthetic constructs known as chimeric antigen receptors (CARs) employ antibody-derived antigen-binding variable heavy and light chain domains and operate in a HLA-independent manner. Genome editing technologies are now being applied to confer additional properties to engineered T cells, with the first clinical applications recently reported. This chapter reviews emerging gene editing tools and then presents the applications of such gene-edited T cells.

## Tools

### Genome Editing Technologies

Engineered nucleases incorporate customizable sequence-specific DNA-binding elements bound to nonspecific cleavage domains [3]. Induction of a nuclease-mediated double-stranded DNA break (DSB) results in the activation of endogenous DNA damage response pathways. Repair occurs in the presence of a suitable DNA template mediated by homology-directed repair (HDR) [4]. In the absence of template, the alternative error-prone nonhomologous end joining (NHEJ) pathway creates insertions or deletions (Indels) at the break point [5]. Indels can yield mRNA transcripts that contain frame-shift and nonsense mutations that undergo degradation through nonsense-mediated decay resulting in disruption of gene function [6]. Four major platforms are currently exploited for site-specific DNA-editing purposes: meganucleases, zinc finger nucleases (ZFNs), transcription activator-like effector (TALE)-nucleases (TALENs), and most recently the clustered regularly interspaced short palindromic repeats (CRISPR/Cas) system.

#### Meganucleases

**Fig. 1:**
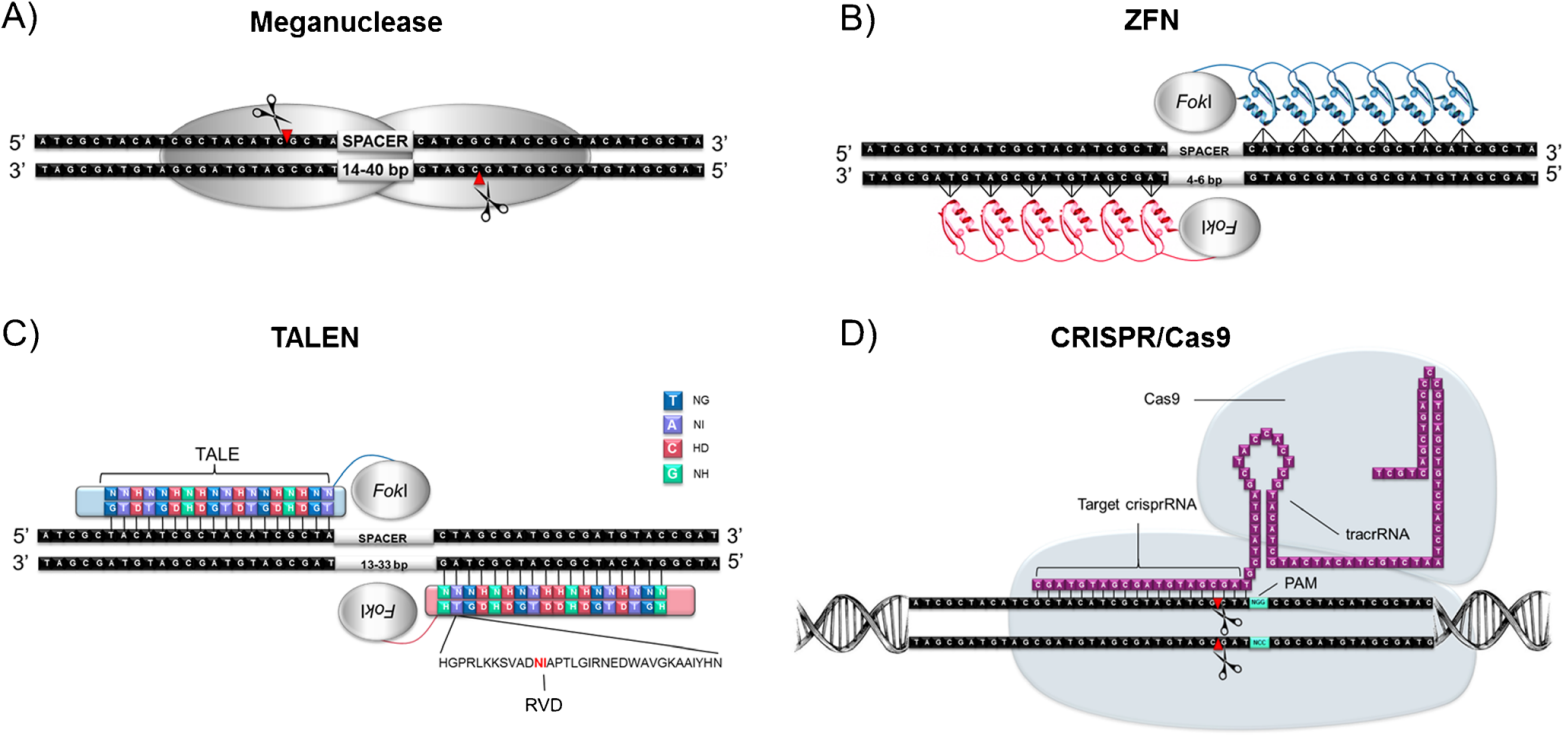
Genome editing technologies. Introduction of double-stranded breaks enables the formation of Indels in the absence of a suitable repair template, leading to knockout of gene function. Several genome editing technologies are currently available, each with a specific mode of action. **a** Meganucleases are homing endonucleases that form dimers in order to cleave. The single nuclease domain is made up of the DNA recognition and cleavage domains. (from Bertoni C. Front. Physiol. 2014, 5:148) [10]. **b** ZFNs require dimerization of two *Fok*1 domains at targeted loci in order for scission to occur. Each zinc finger contacts three nucleotides of the target sequence. (from Didigu CA, Doms RW. Viruses 2012, 4(2), 309–324) [11]. **c** TALEN cleavage is also *Fok*I mediated; however, each TALE contains 34 amino acid repeat sequences, with each RVD targeting a single base in the target sequence. (reprinted by permission from Macmillan Publishers Ltd.: Hyongbum K, Jin-Soo K. Nature Reviews Genetics 2014, 15, 321–334) [12]. **d** CRISPR/Cas9 technology is RNA-guided with Cas9 mediating double-stranded cleavage of the target site. The target site is flanked by a PAM sequence, with double-stranded cleavage occurring three bases upstream from this motif (from Agrotis A, Ketteler R. Front. Genet. 2015, 6:300) [13]

Meganucleases are homing endonucleases derived from I-CreI [7] and I-CeuI [8] and operate as homodimers that recognize DNA sequences with palindromic sequences. Variants containing two motifs, such as I-SceI, act as monomers consisting of a pair of nuclease domains with symmetry-independent targeting [9]. DNA recognition domains have a range of between 14 and 40 bp, leading to double-stranded DNA cleavage, generating 3′ cohesive ends with 4 bp overhangs (Fig. 1a), a feature that may favor recombinogenic HDR compared to other methods (discussed later). This cleavage event initiates the transposition of the endonuclease mobile sequence into the cut site, a mechanism termed “homing” [14]. Site-specific meganucleases have challenging design criteria owing to the single-chained recognition and cleavage domains, although successful modification has been described for a number of applications [15–17]. In the context of gene therapy, meganucleases have for example been modeled for targeted recombination and correction of the RAG1 gene associated with severe combined immunodeficiency (SCID) [18] in hematopoietic stem cells (HSCs) and the XPC gene associated with Xeroderma Pigmentosum in skin cells [19]. Applications within T cell therapies include prevention of graft-versus-host disease (GvHD) via meganuclease-mediated TCR α-chain knockout under conditions for optimal T cell stimulation and meganuclease cleavage efficiency [15]. While engineering of site-specific meganucleases has had some success, their use remains limited within mammalian cells, largely due to complexity of design criteria.

#### Zinc Finger Nucleases

ZFNs are hybrid proteins consisting of a nonspecific *Fok*I cleavage domain and a sequence-specific zinc finger protein that recognizes a predetermined genomic region. The *Fok*I domain requires two DNA-binding events followed by dimerization in order to cleave DNA [20]. Linked to the *Fok*I domain is a zinc finger that consists of approximately 30 amino acids arranged in a ββα configuration. Each zinc finger recognizes approximately 3 bp and binds to the DNA through insertion of the α-helix into the DNA major groove [21] (Fig. 1b). A modular-targeting array binds to specified sequences with high affinity and catalytically induces DNA cleavage and subsequent DNA repair that ultimately results in gene disruption or template-mediated HDR. The specificity of the ZFN may be context-dependent, which adds another level of design complexity [22]. Off-target events are known to occur [23], with obligate heterodimerizing *Fok*1 [20] helping to address this. Examples of future therapeutic applications include Sangamo BioSciences’ targeting of the albumin gene locus in hepatocytes with in vivo applications for the treatment of Hemophilia A, Fabry disease, and Gaucher disease [24]. Trials are in progress for treatment of HIV (NCT01044654 and NCT01252641) and planned for hemophilia B (NCT02695160). Despite some early clinical applications using ZFNs, the difficulty of engineering ZFNs to produce domains with high specificity and affinity may be a limitation for wider deployment.

#### TALENs

TALEs are proteins secreted by bacterial plant pathogens such as *Xanthomonas* and *Ralstonia sp*. In 2009, two independent research groups detailed the mechanism of TALE DNA recognition [25, 26]. They determined that polymorphisms occurred primarily within hypervariable amino acid residues, located at positions 12 and 13 within each tandem repeat. These residues were termed repeat-variable diresidues (RVDs) and corresponded to a single nucleotide target site [25]. Their codes are well-defined, with NN recognizing G or A, NI for A, HD for C, and NG for T [25, 26]. More recently, the RVD NH has also been shown to achieve robust guanine-specific recognition [27]. Further modifications to expand the RVD repertoire have subsequently been developed and have demonstrated substantially reduced off-target cleavage events [28]. The synthesis of a hybrid protein containing the TAL effector fused to the *Fok*I DNA cleavage domain has resulted in the development of TALENs (Fig. 1c).

Advantages of TALENs over ZFNs as a genome editing tool lies in their simpler design criteria compared to de novo synthesis of ZFNs. The lack of recognition code context dependency makes this technology more user-friendly, cost-effective, with more predictable targeting. Additionally, TALENs have demonstrated a higher genome-editing activity [29] while imposing less nuclease-associated toxicity, presumably owed to the lower off-target cleavage affects [30]. TALENs have been substantially utilized in T cell therapies against HIV [31], and virus-specific T cells with resistance to the immunosuppressive effects of corticosteroids have been developed through glucocorticoid receptor knockout [32]. The first clinical application of T cells modified by TALENs has seen successful remission of leukemia in an 11-month-old infant [33••]. The disadvantages of TALENs, however, include the highly repetitive sequence and large size relative to ZFNs which may impede delivery, particularly via size-restricted or reverse-transcribing vectors [34].

#### CRISPR/Cas9

As an alternative to protein-guided methodologies, a number of nucleotide-mediated genome editing techniques have been developed, with the most common used being the CRISPR/Cas9 [35•] system, and more recently CRISPR/Cpf1 [36], and NgArgonaute [37]. While each has their advantages, CRISPR/Cas9 remains the most extensively characterized and widely used system to date. The CRISPR/Cas9 system is a component of the bacterial adaptive immune system used to distinguish between self and nonself. The combination of Cas9 and synthetic guide RNA (gRNA) has been harnessed to provide a two-component programmable system engineered for exploitation in a diverse range of molecular biology applications [38, 39••, 40••]. Cas9 requires formation of a secondary structure via complementary base pairing of the trans-activating (tracrRNA) with the pre-crRNA. This RNA chimera triggers further processing of the crRNA by RNase and induces silencing of foreign DNA by Cas9 [41]. DNA targeting is determined by gRNA complementarity to the genome. The caveat to Cas9 cleavage is that a protospacer adjacent motif (PAM) sequence is required to be juxtaposed to the tracrRNA:crRNA secondary structure on the 3′ end. The *Streptococcus pyogenes* spCas9 consensus PAM sequence is 5′-NGG-3′ which lies adjacent to the region of gRNA complementarity [41]. In the presence of Cas9 and its respective PAM, targeted cleavage of double-stranded DNA occurs between the 3rd and 4th base upstream of the PAM, leading to activation of the DNA damage response pathways [39••] (Fig. 1d). Deactivated Cas9 (dCas9) variants have also been generated through incorporation of inactivating mutations in the Cas9 sequence. Fusions of dCas9 to chromatin modifying activation or repression domains permit regulation of gene expression in the absence of DSBs [42]. The CRISPR approach has risen as the tool of choice for genome editing applications due to its design simplicity, ease of use, and efficacy [43]. Trials of gene edited T cells in lung cancer patients are underway at Sichuan University in Chengdu, China and a US immunotherapy trial using CRISPR-mediated TCR knockout and TCR-modified T cells targeting melanoma, myeloma, and sarcoma has recently been proposed [44].

## Applications

Adoptive immunotherapy relies on the isolation of antitumor lymphocytes, their ex vivo expansion, and subsequent infusion into patients. Naturally occurring tumor lymphocytes, however, have been shown to have weak immunogenicity and limited persistence. Genetic manipulation of T cells to express various tumor-targeting moieties has been extensively used to enhance their antigen avidity, specificity, and downstream effector functions. Harnessing the capabilities of genome editing technologies has further enhanced the scope of T cell therapies, particularly in the development of allogeneic universal T cells and antiviral therapies. Targets, implementation strategies and clinical efficacy of genome-edited T cell therapies shall be discussed in further depth in this section.

### TCR Redirected T Cells

**Fig. 2:**
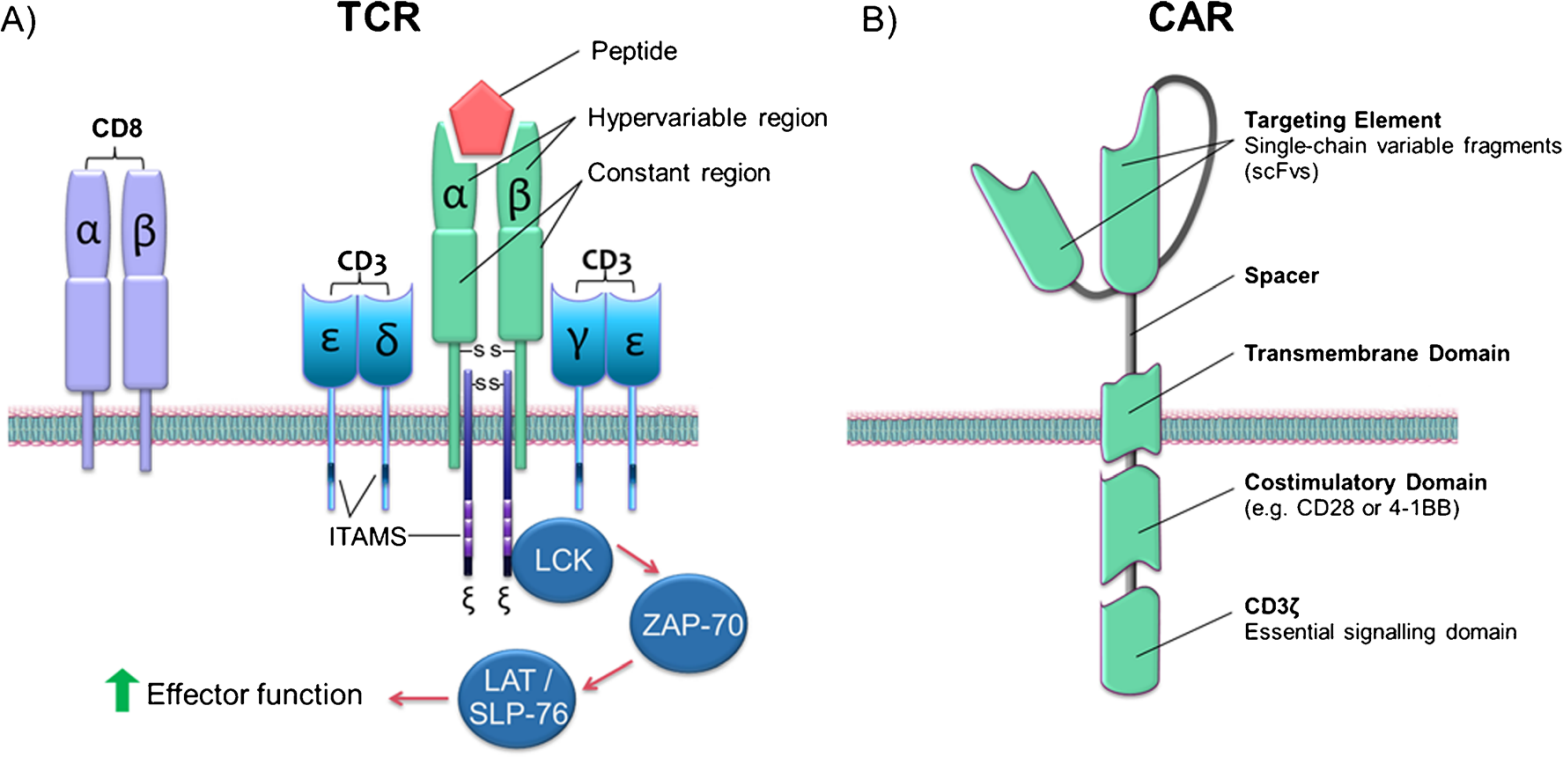
Structure of TCRs and CARs. **a** The TCR is comprised of α and β chains that closely associate with the ε-δ-γ- and ζ-chains of the CD3 complex. Antigen-mediated activation of the α/β chains induces phosphorylation of the ITAMs by LCK. Subsequent activation of ZAP-70 and its downstream targets, LAT and SLP-76, induces an intracellular signaling cascade resulting in the upregulation of genes associated with T cell effector function. (reprinted from Lineberry N, Fathman GC: Immunity 2006, 24(5):501–503, with permission from Elsevier) [46]. **b** Design of the chimeric antigen receptor includes the single-chain variable fragment with antigen-binding affinity, fused to a spacer and transmembrane domain. Effector function is conferred via the TCR CD3ζ domain, while the addition of one (2nd generation) or two (3rd generation) costimulatory domains drives signal activation and amplification of various effector signaling cascades (with permission from Juno Therapeutics: Chimeric Antigen Receptor Technology (CARs) https://www.junotherapeutics.com/our-science/car-technology/) [47]

TCRs detect antigens that have been processed and presented as peptides on cell-surface MHC molecules, inducing activation of T cells in response to antigens. The TCR CD3 complex (CD3ε, CD3δ, CD3γ, and CD3ζ) contains immunoreceptor tyrosine-based activation motifs (ITAMs) that remain dephosphorylated in resting T cells. Upon TCR ligation, the cytoplasmic tails of CD3ε and CD3ζ undergo a conformational change rendering them accessible for phosphorylation via protein tyrosine kinases [45]. ITAM phosphorylation prompts recruitment of complexes involved in organizing effector molecules, allowing accurate spatiotemperal activation of affector signal transduction (Fig. 2a).

Tumor-associated antigens are often expressed during fetal development or at low levels on normal tissues, leading to central tolerance. The first TCR-redirected T cells were against the melanoma antigen, MART-1, which yielded a response rate of 13% [48]. Increased avidity TCRs were subsequently developed for MART-1 and gp100 with reported responses of 30 and 19%, respectively [49]. The limited expression of the cancer-testis antigen, NY-ESO-1, also made this an ideal target antigen. TCRs against this antigen have been trialed in patients with metastatic synovial cell sarcoma (MSCS) and melanoma, with clinical responses reported in 4 of 6 patients with MSCS and 5 of 11 with melanoma, 2 of which demonstrated complete remission after 1 year (NCT00670748) [50]. The June group recently demonstrated sustained antitumor effects and T cell persistence using lentiviral delivery of the NY-ESO-1 targeting TCR within a multiple myeloma phase I/II trial (NCT01352286) [51•]. Also, anti-MAGE-A3 TCR T cells targeting metastatic cancer resulted in clinical regression in 5 of 9 patients; however, there were 2 fatalities and 3 cases of neurological complications, raising concerns for its use in subsequent immunotherapies (NCT01273181) [52].

TCR mispairing, where there is heterologous pairing between the endogenous and recombinant TCRs, is capable of generating novel-targeting specificities and inducing autoreactivity [53]. While codon optimization, murinization of human TCRs, and the addition of cysteine residues have been found to reduce mispairing [54], complete knockout of the endogenous TCR would be advantageous. Further work exploring genome editing to knockout endogenous TCRα/β chains has been achieved using ZFNs [55, 56], TALENs [57, 58], megaTAL, and CRISPR/Cas [59] nucleases. Bonini et al. developed α- and β-chain specific ZFNs against endogenous TCR genes and subsequently introduced the Wilms tumor-1-specific TCR via lentiviral gene transfer. This resulted in high avidity lymphocytes which had reduced alloreactivity but superior expansion and antigen recognition compared to unedited, donor-matched cells that underwent TCR gene transfer alone both in vitro and in NSG mice [56]. Fehse and colleagues performed TCR knockout using TALENs delivered by electroporation of mRNA, resulting in 58 and 41% knockout of the α- and β-chains respectively in primary T cells [57]. Recently, researchers at UPenn gained approval for a phase I trial to generate and transplant autologous T cells devoid of both endogenous TCR and the checkpoint inhibitor, programmed cell death protein-1 (PD-1), using CRISPR, while simultaneously overexpressing a TCR against the NY-ESO-1 peptide [44]. These trials will aim to demonstrate improved biosafety profiles of TCR-edited T cells.

### CAR T Cell Therapies

Tumor-associated target antigens are typically associated with MHCs, restricting T cell therapies to cancers expressing a particular antigen. Furthermore, tumor cells can downregulate HLA class I expression during tumor progression [60]. The need to develop receptors that could target a number of antigens in a MHC-independent manner was critical in the development of the first CAR [61, 62]. Eshhar et al. demonstrated that chimeric T cell receptors in a cytotoxic T cell hybridoma could target in a non-MHC restricted manner giving CAR T cells the ability to directly and potently target tumors which share common target antigens [62].

Structurally, a CAR consists of up to four domains—the antigen recognition domain, a hinge domain, a transmembrane element, and the signaling endodomain (Fig. 2b). The chimeric single chain antibody fragment (scFv) uses a linker to allow self-association of the variable heavy and light chains [63]. Coupling of the scFv to the signal-transducing TCR CD3ζ chain confers therapeutic and potent effector function [64]. Activation of the endodomains induces T cell activation, cytokine release, and non-MHC target cell lysis. Second-generation CARs were constructed to include a co-stimulatory domain arising from the CD28 or 4-1BB domain. Third-generation CARs were configured to express two co-stimulatory domains for enhanced T cell functionality and signal amplification [65].

In brief, the first clinical trials using CAR T therapies were against HIV [66] and subsequently metastatic ovarian [67] and renal cancers [68]. Rosenberg showed proof of clinical efficacy using CARs to treat lymphoma in a single patient in 2010 [69], while June and colleagues reported complete remission of 2/3 patients treated using CARs against chronic lymphocytic leukemia in 2011 [70]. Sadelain et al. later published results demonstrating the antitumor efficacy of a CD19-CAR in 5 patients with relapsed B cell acute lymphoblastic leukemia (B-ALL) [71]. Cooper et al. genetically altered T cells to target B cell malignancies by expressing a CD19-specific CAR delivered by Sleeping Beauty transposition. Infusion of these modified T cells resulted in clinical responses in patients with ALL (*n* = 12) and B cell lymphoma (*n* = 13). This was the first clinical trial to employ the Sleeping Beauty transposon system for the delivery of a CD19-CAR to redirect specificity of isolated T cells [72]. We [73] and others [74] have reviewed trials involving CAR T cell therapies and their outcomes and highlighted key published trials using CAR T cells redirected against CD19 and CD20 (Table 1).

**Table 1:** Published clinical trials using CD19/20 CAR T cells

Target antigen	Target disease	*n*	CAR structure	Delivery method	Origin of T cell	Cell dose	Trial number	Center	Reference
CD19	FL	2	CD3ζ	EP	Autologous	100–2000 × 10^6^/m^2^	NCT00182650	NCI	Jensen (2010) [75]
CD19	FL	1	CD28 + CD3ζ	RV	Autologous	5 × 10^6^/kg	NCT00924326	NCI	Kochenderfer (2010) [69]
CD19	FL; DLBCL	6	CD28 + CD3ζ	RV	Autologous	2–20 × 10^7^/m^2^		BCM	Savoldo (2011) [76]
CD19	CLL; ALL	10	CD28-CD3ζ	RV	Autologous	0.4, 1, 3 × 10^7^/kg over 2 days	NCT01044069	MSKCC	Brentjens (2011) [77]
CD19	CLL	3	4-1BB + CD3ζ	LV	Autologous	0.15–16 × 10^6^/kg	NCT01029366	Upenn	Porter (2011) [78]; Kalos (2011) [70]
CD19	CLL; ALL	9	CD28 + CD3ζ	RV	Autologous	2–30 × 10^6^/kg	NCT00466531	MSKCC	Brentjens (2011) [77]
CD19	FL; CLL; SMZL	8	CD28 + CD3ζ	RV	Autologous	5–55 × 10^6^/kg	NCT00924326	NCI	Kochenderfer (2012) [79]
CD19	ALL	5	CD28 + CD3ζ	RV	Autologous	1.5–3 × 10^6^/kg	NCT01044069	MSKCC	Brentjens (2013) [71]
CD19	CLL; MCL; DLBCL	10	CD28 + CD3ζ	RV	Allogeneic	1–100 × 10^6^/kg	NCT01087294	NCI	Kochenderfer (2013) [80]
CD19	ALL; CLL	8	CD28-CD3ζ	RV	Allogeneic	1.5, 4.5, 12 × 10^7^/m^2^	NCT00840853	BCM	Cruz (2013) [81]
CD19	CLL; ALL; DLBCL; FL; MCL	110	4-1BB-CD3ζ	RV	Autologous	1.5–500 × 10^7^ total cells	NCT01029366	Upenn	Maude (2014) [82•]
CD19	ALL	2	4-1BB-CD3ζ	LV	Autologous	10–100 × 10^6^/kg	NCT01626495	Upenn	Maude (2014) [82•]
CD19	ALL	30	4-1BB-CD3ζ	LV	Autologous	0.76–20.6 × 10^6^/kg	NCT01626495	Upenn	Maude (2014) [82•]
CD19	ALL; CLL	14	CD28-CD3ζ	RV	Autologous	0.2, 1, 2 × 10^8^/m^2^	NCT00586391	BCM	Xu (2014) [83]
CD19	CLL	4	CD28-CD3ζ	RV	Autologous	1–4 × 10^6^/kg	NCT00924326	NCI	Kochenderfer (2015) [84]
CD19	ALL	21	CD28-CD3ζ	RV	Autologous	1, 3 × 10^6^/kg	NCT01593696	NCI	Lee (2015) [85]
CD19	MM	10	4-1BB-CD3ζ	LV	Autologous	1–5 × 10^7^	NCT02135406	Upenn	Garfall (2015) [86]
CD19	NHL	7	CD28-CD3ζ	SBT	Autologous/allogeneic	1 × 10^6^/m^2^	NCT00968760	MDACC	Kebriaei (2016) [87]
		19				1 × 10^6^/m^2^	NCT01497184		
CD19	CLL, SLL, MM	42	4-1BB-CD3ζ	LV	Autologous	1–5 × 10^7/8^	NCT01747486	Upenn	Fraietta (2016) [88]
CD20	MCL; B-NHL	3	CD28–4-1BB-CD3ζ	EP	Autologous	10^8^, 10^9^, 3.3 × 10^9^/m^2^	NCT00621452	FHCRC	Till (2012) [89]
CD20	DLBCL	7	4-1BB-CD3ζ	LV	Autologous	∼0.3–2.2 × 10^7^/kg	NCT01735604	CPLAGH	Wang (2014) [90]

*FL* follicular lymphoma, *MCL* mantle cell lymphoma, *CLL* chronic lymphocytic leukemia, *ALL* acute lymphoblastic leukemia, *BL* Burkitt lymphoma, *DLBCL* diffuse large B cell lymphoma, *HL* Hodgkin’s lymphoma, *NHL* non-Hodgkins lymphoma, *MM* multiple myeloma, *EP* electroporation, *RV* retrovirus, *LV* lentivirus, *SBT* Sleeping Beauty transposition, *BCM* Baylor College of Medicine, *NCI* National Cancer Institute, *FHCRC* Fred Hutchinson Cancer Research Center, *UPenn* University of Pennsylvania, *MSKCC* Memorial Sloan Kettering Cancer Center, *MDACC* MD Anderson Cancer Center, *CPLAGH* Chinese PLA General Hospital

### Gene-Edited CAR T Cells

While CAR T cell therapies have demonstrated immense therapeutic potential, the cost implications and complexity of bespoke T cell therapies for autologous transplantation remains problematic for broader application. The development of a “universal” CAR T cell platform has recently harnessed the gene editing capabilities of TALENs in order to create an “off-the-shelf” adoptive T cell immunotherapy which was validated in vitro [58] and successfully applied to B-ALL [33••]. In order to overcome immune barriers intrinsic to allogeneic transplantations, targeted knockout of the endogenous αβ TCR within donor-derived T cells was performed [91]. In this way, potential induction of GvHD was abrogated. CD52, which is highly expressed on mature B, T, and dendritic cells and is the target of the lymphodepleting CD52 monoclonal antibody, Alemtuzumab, was also targeted for gene disruption [92]. Therefore, CD52 negative cells survive host T cell depletion by Alemtuzumab [91]. Formal clinical trials are now underway in children and adults (NCT02808442, NCT02746952).

Additional targets include the extensively polymorphic HLA region encoding HLA class 1 A, B, C. In the absence of an HLA-identical donor, a HLA-matched unrelated donor may be available [93]. Genetic engineering of HLA expression provides a means of generating allogeneic cells and can be targeted directly (e.g., β2-microglobulin (B2M)) or indirectly via transcription factors and transporter pathways. B2M is required for successful surface assembly of MHC class I molecules [94], with *B2M* knockout resulting in cells devoid of major histocompatibility complex-I (MHC-I) expression [95]. Some groups have endeavored to downregulate the *B2M* and *HLA* loci in primary T cells [96, 97]. However, the posttranscriptional nature of this approach leads to a reduction in antigen levels and not necessarily a complete knockdown. This is problematic as it has been demonstrated that a single peptide-MHC complex can trigger αβ T cell activation and induce the cytolytic response [98]. With the purpose of generating nonself cells that evade host clearance, Torikai and colleagues genetically engineered donor-derived T cells using designer ZFNs to eliminate expression of HLA-A. HLA-A^neg^ cells evaded lysis by HLA-restricted cytotoxic T cells, demonstrating conceptually that these modifications have clinical potential as donor-derived T cells with a disparate HLA repertoire could be administered to multiple recipients [99].

Another target of interest for improved and sustained CAR T cell effectiveness is PD-1. PD-1 is an immune-checkpoint receptor. Its expression is upregulated following T cell activation, limiting T cell effector function, and resulting in T cell exhaustion [100]. Tumor cells upregulate PD-1 ligands leading to diminished immune responses within the tumor microenvironment [101]. As a result, cancer immunotherapeutics have focused on blocking PD-1 activity using monoclonal antibodies [102] and have shown improved efficacy of adoptive CAR T cell therapies [103]. Genome editing however offers a means of permanent deletion of PD-1. This strategy was validated in vitro using primary patient T cells which demonstrated increased IFN-γ production and enhanced cytotoxicity [104]. The June labs successfully edited the HLA, α-, and β-TCR, B2M, and PD-1 loci using CRISPR with 80% gene disruption when co-administered with Cas9 mRNA, and over 70% dual disruption of TCR and HLA-1. These double-negative cells transduced with CD19-CAR demonstrated potent anti-leukemic activity, demonstrating that simultaneous disruption does not affect CAR T cell efficacy. Moreover, these universal T cells exhibited reduced alloreactivity and did not induce GvHD in various NSG mouse models [105]. A trial underway in Chengdu China is using CRISPR to produce PD-1 knockout T cells that are expanded and infused back into patients with resistant and refractory metastatic nonsmall cell lung carcinoma (NCT02793856) [106]. Other proposed trials using CRISPR-mediated PD-1-deficient T cells include those for prostate cancer (NCT02867345), bladder cancer (NCT02863913), and renal cell carcinoma (NCT02867332).

### Genome Editing Targeting HIV

HIV is a single-stranded RNA virus that reverse transcribes RNA to DNA and subsequently integrates into the host genome. It gains cell entry when gp120, an envelope glycoprotein, binds CD4 and co-receptors C-C chemokine receptor 5 (CCR5)(R5), or C-X-C chemokine receptor 4 (CXCR4)(X4). HIV infects a number of immune cells but preferentially replicates within activated CD4^+^ T cells [107]. Over time, there is progressive loss of CD4^+^ T cells due to their destruction, rendering the individual immune compromised. Individuals homozygous for the naturally occurring *CCR5* ^Δ32^ mutation are resistant to HIV infection [108–110], making CCR5 a promising therapeutic target. Transplantation from such a donor has been reported to allow effective HIV eradication in a single case, the “Berlin patient” [111]. ZFNs have been used to knockout CCR5 expression in T cells and mediate HIV resistance in vitro with minimal off-target effects [112, 113]. Mice reconstituted with CCR5-ZFN-modified resting human CD4^+^ T cells, isolated from HIV-1 seronegative individuals, demonstrated resistance when challenged with HIV. Moreover, viral replication was decreased in mice that received CCR5-ZFN-modified T cells of HIV-1 seropositive origin [112]. First-in-human studies assessed the safety of CCR5 ZFNs delivered ex vivo to T cells using adenoviral vector gene transfer [114••]. Infusion of autologous CCR5-ZFN-modified T cells into patients with chronic aviremic HIV isnfection was performed with half the cohort continuing antiretroviral therapy (ART) in conjunction with T cell transfusion, while the remainder underwent an interruption in ART treatment. Modified cells persisted for a mean of 48 weeks and had increased stability compared to unmodified CD4^+^ T cells. CCR5 deletion appears to have conferred a survival advantage to CD4^+^ T cells in individuals infected with HIV and expansion and persistence of modified T cells resulted in improved long-term CD4 counts. Heterozygous CCR5^Δ32^ individuals were able to maintain a viral load of either undetectable or up to 1000 copies/ml during interruption of ART treatment [114••]. Further studies are ongoing including the ZFN-modified autologous HSCs to determine if CCR5 knockout cells can support HIV-protected immunological reconstitution.

TALENs have also been used to edit the CCR5 co-receptor. Mussolina and colleagues compared ZFNs and TALENs targeting the CCR5 locus within HEK293T cells and demonstrated comparable gene disruption (15–30%). However, TALENs exhibited lower cytotoxicity and reduced off-target activity at the CCR2 locus [30]. Mock et al. used mRNA electroporation to deliver a CCR5-TALEN, resulting in HIV resistance in knockout cells. This strategy resulted in targeting efficiencies of >90% in CD4^+^ PM1 cells, commonly utilized in HIV infection assays, and >50% in primary T cells [115]. Others have developed a megaTAL through substitution of the *FokI* catalytic domain for a meganuclease which has high intrinsic affinity and specificity [116]. Adeno-associated virus (AAV) delivery of a GFP-encoding donor template for homology-directed insertion at the CCR5 locus was delivered in conjunction with nuclease mRNA. This resulted in 80% of biallelic alterations and 8–60% HDR into the *CCR5* locus within T cells. Within CD34^+^ HSCs, HDR into the *CCR5* locus resulted in 14% modification offering the possibility of targeted gene insertion of C46, an HIV fusion inhibitor than can confer dual protection against R5 and X4-tropic HIV-1 [117]. Targeting of the CCR5 locus using CRISPR/Cas9 has also yielded robust HIV resistance, with mutation efficiencies ranging from 18 to 74.1% [118–120].

ZFNs specifically targeting the CXCR4 receptor also resulted in T cells resistance to X4-tropic HIV strains in vitro and in vivo, with lower viral titers observed in mice following T cell engraftment [121, 122]. While this would provide clinical benefit, as X4-tropic HIV is associated with greater pathogenicity and positively correlates with progression to acquired immune deficiency syndrome (AIDS), it was shown that CXCR4-ZFN humanized mice lost X4 HIV-1 protectiveness through the emergence of R5-tropic viral mutants [122]. Didigu et al. addressed this issue by simultaneously targeting the CCR5 and CXCR4 coreceptors using ZFNs, resulting in X4- and R5-tropic HIV resistant CD4^+^ T cells in vitro [123]. An alternative approach was demonstrated by Voit et al. utilized ZFNs to introduce three anti-HIV restriction factors, Rev. M10, TRIM5α, and APOBEC3G D128K into the *CCR5* locus, with simultaneous CCR5 knockout and cells exhibited dual R5 and X4 HIV-1 resistance [124]. CRISPR/Cas9 has also been used in studies targeting the CXCR4 locus, with human primary T cells refractory to subsequent HIV-1 infection [125]. Targets downstream of viral entry have also been exploited with LEDGF, the protein involved in viral integration and encoded by *PSIP1*, inactivated by TALENs. Modified Jurkat cells demonstrated inhibited HIV-1 integration and severely impaired viral replication [126].

HIV can remain transcriptionally dormant creating latent HIV reservoirs, with alternative strategies designed to target proviral DNA. Wayengera and colleagues used computational modeling to generate a ZFN that would specifically target an 18 bp sequence within the HIV pol gene or 15 other ZFNs targeting other regions of the proviral genome. Lentiviral delivery resulted in abrogation of pol activity and excision of over 80% of proviral DNA from latently infected cells [127]. The conserved regions of *Gag*, *Pol*, and *Rev.* have been targeted using ZFNs [128]. Others still have targeted the HIV-1 5′ and 3′ long terminal repeat (LTR), excising full-length proviral DNA with an efficiency of 45.9% [129]. Ebina et al. generated two gRNA targets within the TAR sequence of the R-region and within the NF-κB response element in the U3 region. Efficiency was tested on cells transduced with a lentiviral vector encoding TAT-IRES-GFP under transcriptional regulation of the LTR, with mean fluorescence intensity used to determine gRNA efficiency. The authors noted a reduction of GFP^+^ cells from 45.6 to 20% using the TAR-targeting gRNA [130], while Strong et al. used TALENs targeting the same region resulting in 42% cleavage [131]. This proviral targeting strategy was mimicked with both TALENs [132] against integrated lentiviral LTRs and CRISPR/Cas9 in single and multiple configuration [133]. The authors identified targets within the viral U3 LTR that resulted in excision of the entire 9709 bp proviral HIV DNA and prevented reinfection within latently infected T cells [133].

While editing of T cells had shown promise for HIV resistance, two independent publications demonstrated NHEJ-mediated viral escape of Cas9/gRNA suppression [134, 135]. Sequencing of escaped viral mutants identified that the mutations clustered around the gRNA target cleavage site where indels are formed. Destruction of the original guide site yielded novel mutations arising in the viral genome through indel formation, and loss of the guide recognition site rendered CRISPR/Cas therapy ineffective [136]. One alternative to diminish NHEJ-mediated viral escape would be to design multiple target sites within highly conserved regions of the HIV-1 genome. This multiplex approach can yield increased suppression of HIV-1 infection with a decreased GFP intensity, with gRNAs targeted to the LTR shown to be most effective [137]. Finally, recent reports of a designer Brec recombinase efficiently targeting and excising LTRs on scale holds promise for tackling latent proviral HIV [138].

### Gene Editing Inherited T Cell Defects

Primary immunodeficiencies (PIDs) are model disorders for treatment by HSC transplantation and gene therapy although current “gene-addition” approaches have been associated with insertional mutagenesis in some conditions, following viral integration near *LMO2* proto-oncogenes, among others, resulting in malignancy [139]. Gene-editing approaches should eventually allow in situ gene repair and stem cell-derived reconstitution for SCID disorders and other PIDs. In the first instance, gene repair of T cells in conditions where cells are present but functionally impaired is being investigated, given the efficiency of editing reagents in these cells. One such condition is HyperIgM syndrome or CD40 ligand deficiency. This defect in cellular immunity renders individuals particularly susceptible to opportunistic infections [140]. Antigen presentation initiates the upregulation and expression of CD40L with its expression finely regulated and activation state dependent. A primary function of CD40L is to convey activation signals to B cells [141]. Mutations within *CD40L* result in an inability to undergo immunoglobulin class switching, the mechanism used in B cells to switch from the production of one immunoglobulin to another [142]. TALEN editing of *CD40L* has yielded successful gene correction within primary T cells via homology-directed insertion of a recombinant transgene. Inclusion of the region upstream of Exon 1 permitted the maintenance of endogenous regulation, while inclusion of the 3′-UTR conserved posttranscriptional regulation. Edited cells mimicked the response of wild-type T cells in their activation response, while rescuing the class-switching capabilities of B cells in vitro [143]. Kuo et al. have used CRISPR/Cas9 technology to target patient-specific splice-site mutations within exon 3 of *CD40L*. A template containing a unique restriction enzyme site was co-electroporated into K562 cells, with HDR confirmed via restriction digest [144]. Other disorders with similar T cell defects could also be targeted by a similar approach and offer a route to therapy.

While most applications of genome editing rely on gene knockout or HDR, these technologies are also amenable to gene repair without induction of double-stranded DNA breaks; a technology termed “base editing”. Komor and colleagues demonstrated that merging the guide RNA-mediated targeting ability of dCas9 or Cas9 nickase to the cytidine deaminase enzyme, APOBEC1, results in the ability of the enzyme to convert cytidine to uridine in a site-specific manner [145]. Single-base editors are particularly attractive for permanent correction of diseases caused by point mutations, such as HIGM1, and broaden the scope of genome editing technologies for clinical applications.

## Conclusions

Genome editing modalities have emerged as fundamental tools with which to modify gene expression in a highly specific manner. Recent clinical successes have shown the utility of genome-edited cell-based therapies, undoubtedly an impetus to treat a wider variety of conditions in the future. Improved genome-editing specificity and design of superior genome editing systems will enhance the translational potential and safety profiles of future T cell-based therapies, further driving the transition from research to clinical translation.
